# Use of BioMime Morph stent in treating left main triple vessel disease: a case report

**DOI:** 10.1186/s43044-020-00081-1

**Published:** 2020-08-04

**Authors:** Yash Paul Sharma, Krishna Santosh, Prashant Panda, Krishna Prasad, Lipi Uppal, Shrimanth Y S, C. R. Pruthvi, B. Dinakar

**Affiliations:** Department of Cardiology, Post Graduate Institute of Medical Education and Research, Chandigarh, India

**Keywords:** BioMime Morph stent, Left main disease, Chronic total occlusion

## Abstract

**Background:**

Diffuse long coronary lesions require long overlapping stents which produce less than optimal long-term results. Sizing of long stents becomes difficult owing to tapering of coronaries and overlapping with excessive metal which makes restenosis a nagging problem on long-term follow-up. The optimal stent sizing becomes even more important when left main (LM) needs to be treated along with left ascending artery (LAD) or left circumflex artery (Lcx). The chronic total occlusions (CTO) represent other complex diffuse coronary lesions which not only require higher expertise and better hardware but also usually long lengths of overlapping stents. The long-tapered sirolimus-eluting stent system (BioMime Morph) has been successfully used in long diffuse lesions in individual coronaries including CTO but the use of the same in LM-LAD/LM-Lcx diffuse lesions has not been explored well where its tapered design can really be favourable.

**Case presentation:**

We here present a case of a 51-year-old hypertensive male presented with NSTEMI and angiography showing left main triple vessel disease with CTO of right coronary artery (RCA). We successfully stented the LM-LAD and RCA (staged) using a long-tapered BioMime Morph system. IVUS was used for optimising the LM-LAD stent. At 6 months follow-up, the patient was doing well on double anti-platelets.

**Conclusion:**

Complex coronary disease, involving the left main and LAD diffusely and CTO of RCA, can be well managed by using a single long-tapered stents thereby avoiding multiple stenting strategy. The stents with decremental diameter will provide better adaptation to the vessel size and their natural tapering. The usage of intravascular imaging helps in better optimisation of stents

## Background

Diffuse long coronary lesions require long overlapping stents which produce less than optimal long-term results. Sizing of long stents becomes difficult owing to tapering of coronaries and overlapping with excessive metal which makes restenosis a nagging problem on long-term follow-up. The optimal stent sizing becomes even more important when left main (LM) needs to be treated along with left ascending artery (LAD) or left circumflex artery (Lcx). The chronic total occlusions (CTO) represent other complex diffuse coronary lesions which not only require higher expertise and better hardware, but also usually long lengths of overlapping stents. The long-tapered sirolimus-eluting stent system (BioMime Morph) has been successfully used in long diffuse lesions in individual coronaries including CTO but the use of the same in LM-LAD/LM-Lcx diffuse lesions has not been explored well where its tapered design can really be favourable.

## Case presentation

A 51-year-old hypertensive male presented to the cardiac critical care unit with a history of recurrent episodes of acute onset retrosternal chest pain at rest for the last 2 days. He was admitted in view of ongoing chest pain associated with diaphoresis. On admission, the patient was tachypneic with a respiratory rate of 22 breaths/min, the temperature of 37.1 °C, blood pressure was 140/96 mmHg, heart rate was 102 beats/min, oxygen saturation was 96%, and oxygen was 4 l/min. On auscultation, lungs were normal, and the cardiovascular system had tachycardia with no audible murmurs. Cardiac biomarkers revealed elevated troponin 1.1 ng/ml. ECG showed tachycardia and normal sinus rhythm with dynamic changes. Transthoracic echocardiography showed wall motion abnormalities in LAD and RCA territory with an ejection fraction of 44%. He was diagnosed as acute coronary syndrome, non-ST segment elevation myocardial Infarction (NSTEMI).

## Procedure details

**Fig. 1:**
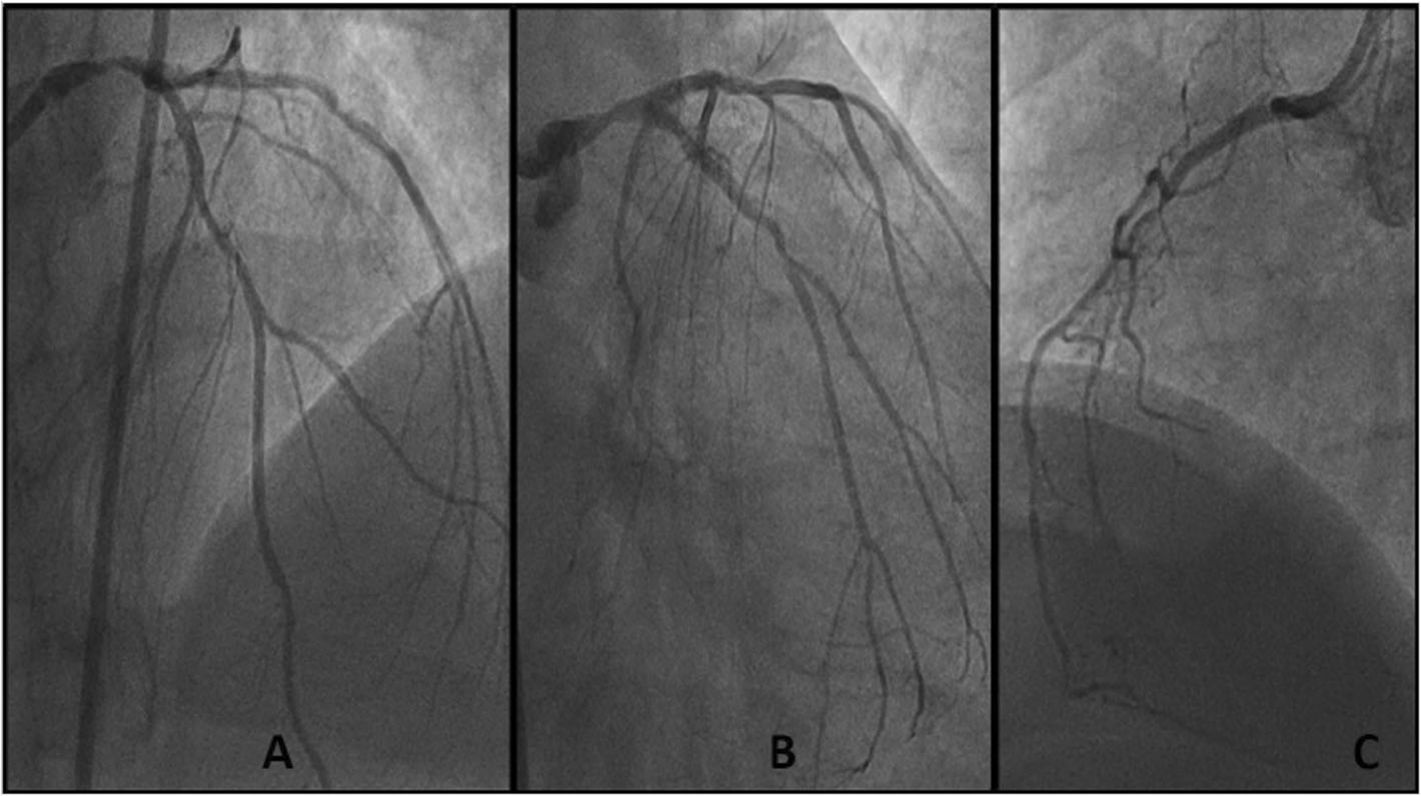
Diagnostic angiogram **a** showing distal LM stenosis of 85 to 90% followed by diffuse mid LAD stenosis of 75 to 80% (**b**). Proximal LCX stenosis of 55 to 60% (**c**). Proximal diffuse RCA stenosis of 75 to 80% followed by CTO

**Fig. 2:**
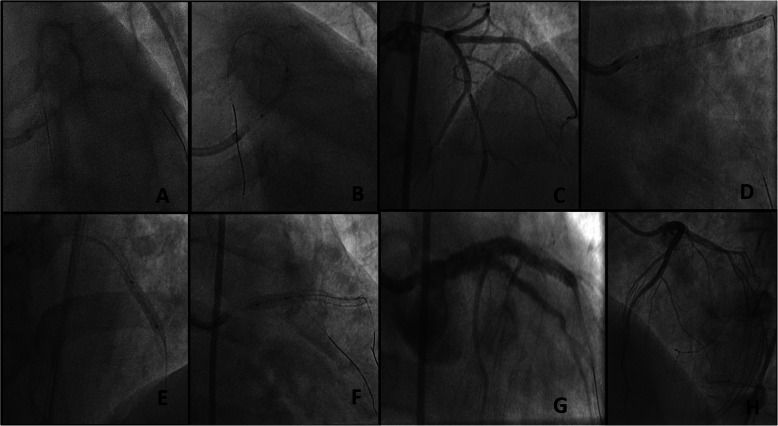
Images showing percutaneous coronary intervention procedure in LM-LAD (**a**). Wiring both LAD and LCX arteries (**b**). Balloon dilation of LM lesion (**c**). Balloon dilation of LAD lesion (**d**). DES BioMime Morph 3.5–3.0 × 60 mm in deployed LM-LAD (**e**). Post dilation in LAD (**f**)—POT in LM (**g**, **h**). Final result with TIMI III flow

**Fig. 3:**
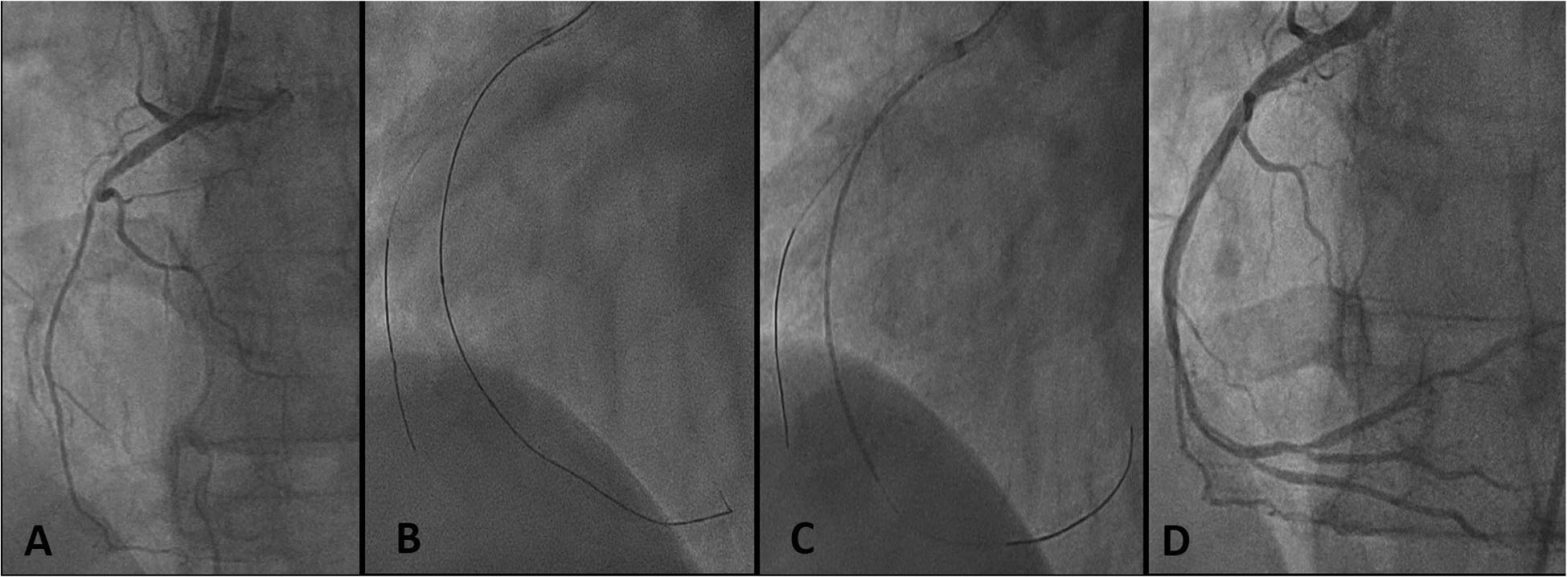
Images showing percutaneous coronary intervention procedure in RCA (**a**). Proximal diffuse RCA stenosis of 75 to 80% followed by CTO (**b**). Wiring of the RCA (**c**). DES BioMime Morph 3.0–2.5 × 60 mm placed in RCA (**d**). Final result with TIMI III flow

The patient underwent emergency angiography using the right femoral approach. Diagnostic angiography revealed left main distal 70% stenosis and diffusely diseased LAD, a borderline disease in the left circumflex artery and cut off of right coronary artery with a SYNTAX (Synergy between PCI with Taxus and Cardiac Surgery) score of 33 as shown in Fig. 1. He was advised coronary artery bypass grafting (CABG), but eventually taken for percutaneous coronary intervention in view of ongoing pain and unwillingness for CABG by the patient and attendants. Percutaneous coronary intervention (PCI) was performed using the right femoral artery with a 7-Fextra back up guiding catheter. Fielder (ASAHI Intecc, Aichi, Japan) wire was used to access the left anterior descending artery and Sion blue (ASAHI Intecc, Aichi, Japan) wire was used to access the left circumflex artery (Fig. 2). In view of calcification, the lesion was predilated with ACCUFORCE^TM^ (TERUMO) 2.5 × 20 mm non-compliant (NC) balloon in left main and LAD. Initially thought of going for 2 separate overlapping stents, but later it was decided to go for a single long stent which is tapered such that we can avoid extra metallic frames in the vessel. A BioMime Morph 3.5–3.0 × 60 mm sirolimus-eluting coronary stent (Meril Life Sciences, Vapi, Gujarat, India) was used covering both the left main and LAD (off label). The stent was deployed at 10 atm pressure and the lesion was post dilated with 3.5 × 20 mm NC balloon. Post dilation intravascular ultrasonography (IVUS) was done which revealed diameters of left main and LAD size of 4.69 mm and 3.97 mm, respectively. Stent in LAD was post dilated with 4 × 12 mm NC balloon and stent in left main was post dilated with 4.5 ×°12 mm and 5 × 8 mm NC balloon. Post dilation IVUS showed a well-apposed stent with TIMI 3 flow in LAD. The mean fluoroscopy time was 16 min 29 s and the total cumulative air kerma product was 1.8 Gy. He was kept on aspirin and ticagrelor. He underwent staged PCI to RCA 1 week later which was chronic total occlusion (CTO) (Fig. 3). PCI was performed using the right femoral access and 6-F JR 3.5 guiding catheter was used. FIELDER XT-A (ASAHI Intecc, Aichi, Japan) wire was used to access the right coronary which was successful and lesion was predilated with RYUREI^TM^ (TERUMO) 1.0 × 5 mm NC balloon and later with 2.5 × 20 ACCUFORCE^TM^ (TERUMO) NC balloon. In view of diffusely diseased RCA, a single long-tapered stent BioMime Morph 3.0–2.5 × 60 mm was deployed in proximal to distal RCA, the lesion was post dilated with 3 × 20 mm NC balloon. Final angiography demonstrated thrombolysis in myocardial infarction (TIMI 3) flow in RCA. Repeat echocardiography demonstrated a LV ejection fraction of 55%. He was discharged a day later and is hemodynamically stable on regular follow-up for the last 6 months.

## Discussion

For optimal results of PCI, sizing of stents is critically important particularly for lesions involving the vessels with different diameters. Treatment of long segment CTO or tandem lesions requires the usage of 2 or more overlapping stents, which are associated with increased neointimal proliferation, increased risk of stent fracture, vascular injury, and in-stent restenosis. An increased layer of stent struts is also associated with higher rates of revascularization [1, 2]. Oversizing and under-sizing of stents may lead to stent thrombosis and increased risk of in-stent restenosis [3]. Coronary arteries show a natural taper in their course. Tapering is defined as the ratio of the area change to the vessel length [4]. A study by Zhang et al. [5] in Asian adults showed a decremental ratio of 7.7% and 5.1% in LAD and RCA vessels between the proximal and distal end. Therefore, it is challenging to decide the optimal sizing of stents, particularly for long segment lesions. Tapered stents mitigate the complications and maximise the clinical benefit in this kind of diffuse discrepant lesions. The long-tapered BioMime Morph stents have a peculiar feature of morphology mediated expansion, thereby preventing artery dissection and over-inflation at the distal end of the stent. The BioMime Morph is a biodegradable polymer-based sirolimus-eluting novel tapered stent with the ultra-thin strut of 65 m of cobalt chromium platform. Studies have shown that the usage of tapered stents in daily clinical practice showed good apposition and coronary flow without any complications [6, 7]. Very few cases documented the feasibility of using long-tapered stents in diseases involving the left main [6]. Here, we report the case of complex triple vessel disease with left main involvement and CTO of RCA which was managed successfully with long-tapered BioMime Morph stents.

## Conclusion

With this case, we opine that complex coronary disease involving the left main and LAD diffusely and CTO of RCA can be well managed by using single long-tapered stents thereby avoiding multiple stenting strategy. The stents with decremental diameter will provide better adaptation to the vessel size and their natural tapering. The usage of intravascular imaging helps in better optimisation of stents. To our knowledge, this is the first case where left main and RCA CTO were managed successfully with BioMime Morph stent in the same patient and in index hospitalisation.

## Data Availability

The datasets used and/or analysed during the current study are available from the corresponding author on reasonable request.

## Abbreviations

LM: Left main
LAD: Left ascending artery
Lcx: Left circumflex artery
CTO: Chronic total occlusions
NSTEMI: Non-ST segment elevation Myocardial Infarction
TIMI: Thrombolysis in myocardial infarction
PCI: Percutaneous coronary intervention
IVUS: Intravascular ultrasonography
NC: Non-compliant
SYNTAX: Synergy between PCI with Taxus and Cardiac Surgery
CABG: Coronary artery bypass grafting
